# The effect of spironolactone on diastolic function in haemodialysis patients

**DOI:** 10.1007/s10554-021-02176-5

**Published:** 2021-02-05

**Authors:** T. Hauser, V. Dornberger, U. Malzahn, S. J. Grebe, D. Liu, S. Störk, M. Nauck, N. Friedrich, M. Dörr, C. Wanner, V. Krane, F. Hammer, for the MiREnDa Study Group, Collaborating investigators (sites), Susanne Berweck, Patrick Biggar, Christoph Blaser, Thomas Bochannek, Frank Breunig, Michael Brunner, Beatrix Büschges-Seraphin, Stefan Büttner, Ahmet Cakmak, Thomas Döltz, Mara Dörken, Kai-Uwe Eckardt, Heribert Fink, Stefan Fischer, Wolfgang Freisinger, Tilo Freiwald, Julian Gebhardt, Helmut Geiger, Rüdiger Götz, Jan Goßmann, Renate Hammerstingl, Joanna Harazny, Michael Heckel, Andrea Heyd-Schramm, Joachim Hoyer, Rolf Janka, Oliver Jung, Markus Ketteler, Christina Klaeffling, Claudius Kleinert, Marianne Kleinert, Arnfried Klingbeil, Thorsten Klink, Benjamin-Florian Koch, Judith Kosowski, Michael Leidig, Jens Lutz, Mohamed Marwan, Maria Moritz, Brigitte Moye, Holger Naujoks, Kai-Olaf Netzer, Ulrike Raff, Clemens Reichert, Imke Reimer, Jurij Ribel, Sophie Richter, Christian Ritter, Sarah Rudolf, Beate Schamberger, Michael Schmid, Thomas Schmiedeke, Andreas Schmitt, Heike Schneider, Reinhard Schneider, Cord Schneuzer, Markus Schöffauer, Lothar Schramm, Sabine Schütterle, Susanne Schwedler, Ewelina Sobkowiak, Daniel Sollinger, Frank Strutz, Sebastian Toncar, Vladimir Vasiljuk, Thomas Vogl, Thorsten Walther, Julia Weinmann-Menke, Bettina Wirth, Hendrick Witsch, Paul Würmell, Raoul Zeltner, Josef Zimmermann

**Affiliations:** 1 Division of Nephrology, Department of Medicine I, University Hospital Würzburg, Oberduerrbacher Str. 6, 97080 Wuerzburg, Germany; 2 Department of Internal Medicine B, University Medicine Greifswald, Greifswald, Germany; 3 Clinical Trial Centre, University Hospital Würzburg, Würzburg, Germany; 4 Department of Paediatrics, University Hospital of Erlangen, Erlangen, Germany; 5 Comprehensive Heart Failure Centre, University and University Hospital Würzburg, Würzburg, Germany; 6 Institute for Clinical Chemistry and Laboratory Medicine, University Medicine Greifswald, Greifswald, Germany; 7 German Centre for Cardiovascular Research (DZHK), partner site Greifswald, Greifswald, Germany

**Keywords:** Diastolic function, Echocardiography, E/e’, Haemodialysis, HFpEF, Spironolactone

## Abstract

Heart failure with preserved ejection fraction (HFpEF) is highly prevalent in patients on maintenance haemodialysis (HD) and lacks effective treatment. We investigated the effect of spironolactone on cardiac structure and function with a specific focus on diastolic function parameters. The MiREnDa trial examined the effect of 50 mg spironolactone once daily versus placebo on left ventricular mass index (LVMi) among 97 HD patients during 40 weeks of treatment. In this echocardiographic substudy, diastolic function was assessed using predefined structural and functional parameters including E/e’. Changes in the frequency of HFpEF were analysed using the comprehensive ‘HFA-PEFF score’. Complete echocardiographic assessment was available in 65 individuals (59.5 ± 13.0 years, 21.5% female) with preserved left ventricular ejection fraction (LVEF > 50%). At baseline, mean E/e’ was 15.2 ± 7.8 and 37 (56.9%) patients fulfilled the criteria of HFpEF according to the HFA-PEFF score. There was no significant difference in mean change of E/e’ between the spironolactone group and the placebo group (+ 0.93 ± 5.39 vs. + 1.52 ± 5.94, p = 0.68) or in mean change of left atrial volume index (LAVi) (1.9 ± 12.3 ml/m^2^ vs. 1.7 ± 14.1 ml/m^2^, p = 0.89). Furthermore, spironolactone had no significant effect on mean change in LVMi (+ 0.8 ± 14.2 g/m^2^ vs. + 2.7 ± 15.9 g/m^2^; p = 0.72) or NT-proBNP (p = 0.96). Treatment with spironolactone did not alter HFA-PEFF score class compared with placebo (p = 0.63). Treatment with 50 mg of spironolactone for 40 weeks had no significant effect on diastolic function parameters in HD patients.

The trial has been registered at clinicaltrials.gov (NCT01691053; first posted Sep. 24, 2012).

## Introduction

Before entering haemodialysis (HD) treatment, 87% of chronic kidney disease (CKD) patients already present with at least one echocardiographic abnormality (e.g. left ventricular hypertrophy, diastolic dysfunction) reflecting structural and functional impairment of the heart [1]. In particular, heart failure with preserved ejection fraction (HFpEF) is highly prevalent among patients with end-stage kidney disease (ESKD) [2] and its manifestations like an increased left atrial volume index (LAVi) or increased E/e’ ratio were associated with a higher risk of cardiovascular events in ESKD patients [3]. Aldosterone plays a key role in the development of myocardial fibrosis, endothelial dysfunction and blood pressure management which are important factors in the genesis of HFpEF and by counteracting its effects, mineralocorticoid-receptor antagonists (MRA) like spironolactone reduce fibrosis in myocardial tissue and improve myocardial stiffness which has been associated with ameliorated diastolic function in past trials [4–9]. MRA were repeatedly investigated as a potential treatment for HFpEF, however, results regarding efficacy were inconclusive [10–12].

Diagnosing HFpEF in ESKD patients is difficult because even well-established classification systems like the New York Heart Association (NYHA) functional classes have their limitations when applied to HD patients. In most cases, the observed dyspnoea rather reflects a patient’s actual fluid status than cardiac impairment [13]. Additionally, regardless of myocardial dysfunction, NT-proBNP serum levels are markedly higher in HD patients due to intermittent volume overload between HD sessions [2]. There is no dedicated diagnostic guideline for this patient group and most HFpEF trials excluded patients with higher degrees of renal impairment. According to current guidelines, echocardiography is an established tool in the diagnostics of diastolic function [14, 15] and septal E/e’ appears to be the most suitable individual parameter to evaluate left ventricular diastolic function in ESKD patients [16, 17] and was found to predict hospitalization in this collective [18]. Furthermore, in 2019, the European Society of Cardiology introduced the ‘Echocardiographic and natriuretic peptide score’ as part of the Heart Failure Association ‘HFA-PEFF diagnostic algorithm’ (here termed ‘HFA-PEFF score’) in its latest consensus recommendation creating a new tool to identify HFpEF patients [15]. Using these tools, we investigated the efficacy of spironolactone to improve echocardiographic parameters of diastolic function and report on cardiac structure and function with specific focus on diastolic function parameters based on data from the MiREnDa trial.

## Materials and methods

### Study design

The MiREnDa trial was a multi-centre, randomized, double-blind, placebo-controlled study to investigate cardiovascular efficacy and safety of spironolactone in HD patients. The detailed study design has been published previously [19]. In brief, participants were randomized 1:1 to 50 mg spironolactone once daily or placebo and treated for 40 weeks. Major eligibility criteria comprised an age ≥ 18 years and maintenance HD, whereas exclusion criteria were MRA treatment during the past six months, history of hyperkalaemia (serum potassium ≥ 6.5 mmol/l) and arterial hypotension. The primary endpoint of the trial was change in left ventricular mass index (LVMi) measured by cardiac magnetic resonance (CMR) imaging. Parameters of diastolic function and biomarker workup were secondary endpoints. Patients were enrolled at 20 dialysis centres and cardiovascular assessment was performed in one of the three participating university centres (Frankfurt, Erlangen-Nuremberg, and Würzburg) according to predefined standardized procedures. During the MiREnDa study visits, trained personnel used standardized questionnaires to obtain information on the patient´s medical history, sociodemographic factors and medication intake. Comorbidities were extracted from charts provided by general practitioners and nephrologists. All clinical measurements were performed according to predefined standard operating procedures. The study was approved by the Ethics-committee at the Medical Faculty of the University of Würzburg and performed according to the International Conference on Harmonisation Good Clinical Practice guidelines and the Declaration of Helsinki. All study participants provided written informed consent before study entry. The trial has been registered at clinicaltrials.gov (NCT01691053; first posted Sep. 24, 2012).

### Cardiac Imaging

Echocardiographic data were acquired according to a standardized protocol and analysed in a core lab at the University Hospital Würzburg, Germany. The investigators were blinded to treatment group assignment. Prior to patient enrolment, all study sites (University Hospitals Würzburg, Erlangen-Nürnberg, Frankfurt) submitted test recordings which were subsequently evaluated by the MiREnDa core lab to ensure image quality. In order to minimize variations in fluid status and to ensure comparable loading conditions, echocardiography was scheduled on dialysis free days. The acquired data met the standards proposed by Nagueh et al*.* [14]. Assessment of diastolic function included early peak mitral annular tissue velocity (e’ in cm/s) in the 4 chamber long-axis view with tissue doppler imaging. Early (E) and late (A) peak trans-mitral flow rate (in ml/s) and E/A ratio were analysed using pulsed wave (pw) doppler. E deceleration time (E-DT) was measured from peak E velocity to baseline. Global longitudinal strain (GLS) was assessed semi-automatically using GE Healthcare EchoPAC software (version 202, GE Healthcare, Chalfont St Giles, UK). For isovolumetric relaxation time (IVRT) measurement, the continuous wave (cw) doppler sample was placed between the aortic valve and anterior mitral valve leaflet in order to assess the time between aortic valve closure and mitral inflow. For E/e’, septal e’ was used. Left ventricular mass (LVM) was calculated according to the American Society of Echocardiography 2005 guidelines [20] and normalized to body surface area utilising the Mosteller formula [21]. Left ventricular hypertrophy (LVH) was defined as an LVMi > 115 g/m^2^ in men and > 95 g/m^2^ in women [20]. Left atrial volume was calculated according to the 2005 ASE recommendation for chamber quantification using images of the four chamber view and two chamber view acquired by CMR imaging (biplane area-length method) [20] and normalised to body surface area with the Mosteller formula [21]. To interpret left atrial volume index (LAVi) size, we categorized the obtained data in four categories using CMR-specific cut-off values proposed by Khan et al. [22]; normal (21–52 ml/m^2^), mildly (52–62 ml/m^2^), moderately (63–73 ml/m^2^) and severely enlarged (> 73 ml/m^2^). In analogy to the LAVi cut-off values for echocardiographic measurements proposed by the ASE recommendation for chamber quantification [20] and the European Society of Cardiology 2019 consensus recommendation [15], we defined all LAVi values exceeding 62 ml/m^2^ as relevantly enlarged. Clinical figures were designed using Adobe Creative Suite 6 Photoshop (Adobe Systems, San Jose, USA).

### Measurement of NT-proBNP

At enrolment and follow-up, biomaterials were collected in a standardised fashion, frozen at −80 °C and transported to the central biobank for future analysis. NT-proBNP was measured in serum samples in a central certified laboratory (Institute for Clinical Chemistry and Laboratory Medicine, University Medicine Greifswald, Greifswald, Germany) using the Siemens Dimension Vista® System (PBNP Flex® reagent cartridge, Cat. No. K6423A, Siemens Healthcare Diagnostics Ltd., UK).

### Definition of HFpEF

All patients were evaluated by the composite score of the Heart Failure Association ‘HFA-PEFF diagnostic algorithm’ according to the European Society of Cardiology 2019 consensus recommendation. The HFA-PEFF score is composed of a functional, a morphological and a biomarker domain [15]. In each domain, two points are granted per ascertained major criterion and one point per minor criterion with a maximum of two points per domain resulting in a span width of zero to six points. A score of five or more points in combination with a preserved LVEF > 50% was suggestive of HFpEF, whereas the presence of HFpEF was regarded highly unlikely with a score of one or lower. For patients receiving a score between two and four points, the HFA-PEFF score suggests further testing to confirm the presence or absence of HFpEF. We considered septal e’, tricuspid regurgitation peak velocity, septal E/e’, LAVi, LVMi, GLS, left ventricular wall thickness and relative wall thickness as well as rhythm-specific NT-proBNP (applying different cut-off values depending on the presence of sinus rhythm or atrial fibrillation) for calculation of the HFA-PEFF score. As our LAVi measurements were based on CMR imaging instead of echocardiography, we used adjusted cut-off values to apply our data on the HFA-PEFF score (major criterion: > 62 ml/m^2^; minor criterion: 52–62 ml/m^2^). Changes in HFpEF status over time were analysed according to the three categories “no change”, “deterioration” and “improvement” depending on an observed shift between the three HFA-PEFF score classifications “no HFpEF” (0–1 points), “further testing required” (2–4 points) and “HFpEF” (5–6 points). “Deterioration” indicated an increase in the HFA-PEFF score above the mark of five points, “improvement” a decrease below the mark of five points and “no change” indicated the persistence of a score either below or above the mark of five points.

### Statistical analysis

Our research hypothesis was that 50 mg of spironolactone administered once daily had an effect on E/e’ measurements and other cardiac function parameters in HD patients and would subsequently be reflected in a reduction of patients with a HFA-PEFF score of five or higher. Sample distributions of baseline characteristics were summarized by appropriate descriptive statistics per study groups. We screened echocardiographic measurements at baseline for extreme values, defined as values twofold the interquartile range above the third or below the first quartile. We performed one-way ANCOVA analyses with adjustment for the respective baseline values to compare the mean change of E/e’ and all assessed diastolic function parameters from baseline to a nine-month follow-up between the two treatment groups. The impact of MRA administration on HFA-PEFF score classifications was investigated using the Freeman-Halton test. We did not used methods to adjust for multiple comparisons, thus all p-values are exploratory under the assumption of a significance level of p < 0.05. All statistical analyses including figures were executed using IBM SPSS Statistics (versions 25 and 26, IBM, Armonk, USA).

## Results

**Fig. 1:**
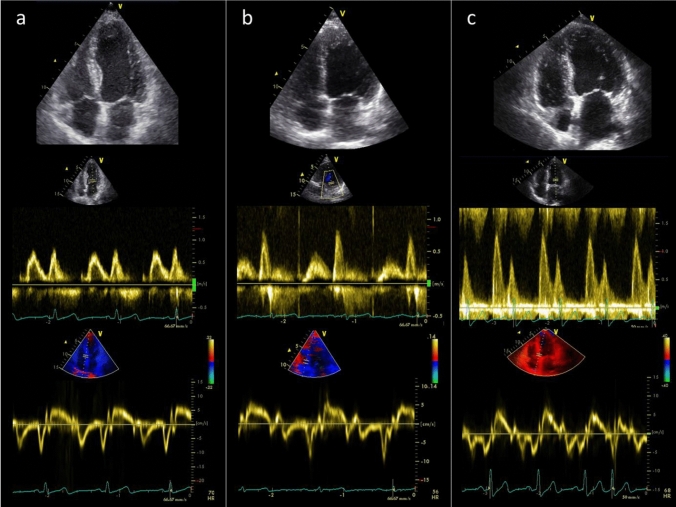
Echocardiographic assessment of cardiac remodelling and diastolic dysfunction in three exemplary female ESKD patients with preserved ejection fraction. Upper, middle, and lower panels respectively show apical 4-chamber view (upper panel), pulsed-wave Doppler evaluated diastolic filling pattern (middle panel), and tissue Doppler early diastolic mitral annular velocity (e´, lower panel). **a** Apparently healthy heart, LVMi = 59 g/m^2^, LVEF = 72%, LAVi = 33 ml/m^2^, E/e’ = 8.9 **b** Beginning LV hypertrophy and mild diastolic dysfunction, LVMi = 103 g/m^2^, LVEF = 61%, LAVi = 53 ml/m^2^, E/e’ = 12.6 **c** Severely enlarged LV and severely impaired diastolic function, LVMi = 147 g/m^2^, LVEF = 67%, LAVi = 56 ml/m^2^, E/e’ = 33.0

Of the 97 patients included in the MiREnDa trial, full echocardiographic assessment was available in 65 patients (59.5 ± 13.0 years, 21.5% female) exhibiting a preserved systolic left ventricular function (LVEF ≥ 50%). Comparisons of summary statistics for baseline characteristics between both study groups indicated that randomization had worked well (Table 1). At baseline, mean E/e’ was 15.2 ± 7.8 (interquartile range (IQR): 10.1–18.8; percentage of extremes: 1.5%), mean LAVi was 43.2 ± 17.8 ml/m^2^ (IQR: 29.7–54.0 ml/m^2^; percentage of extremes: 1.5%) and 37 patients (56.9%) scored five or more points in the HFA-PEFF score suggestive for HFpEF; mean LVMi was 92.8 ± 23.7 g/m^2^ (IQR: 72.2–104.9 g/m^2^; percentage of extremes: 1.5%) and 11 (16.9%) patients presented with left ventricular hypertrophy (LVH) (Fig. 1).

**Table 1:** Baseline characteristics

Characteristic	Placebo (n = 34)	Spironolactone (n = 31)
Age (years)	59.4 ± 12.7	59.5 ± 13.5
Female, n (%)	7 (20.6)	7 (22.6)
NYHA functional class, n (%)		
I	19 (55.9)	16 (53.3)
II	11 (32.4)	6 (20.0)
III	4 (11.8)	8 (26.7)
Left ventricular hypertrophy, n (%)	5 (14.7)	6 (19.4)
Heart rate, min^−1^	70.6 ± 10.3	70.9 ± 10.3
Systolic blood pressure, mmHg	136.4 ± 22.1	139.3 ± 19.0
Diastolic blood pressure, mmHg	82.5 ± 15.1	81.6 ± 11.7
BMI, kg/m^2^	27.3 ± 5.2	28.7 ± 5.3
Time on dialysis, months	45.0 (7.8–82.0)	37.0 (18.0–88.0)
NT-proBNP, pg/ml	2877 (1086–6425)	3267 (1447–11,486)
Comorbidities		
Arterial hypertension, n (%)	33 (97.1)	28 (90.3)
Atrial fibrillation, n (%)	3 (8.8)	3 (9.7)
COPD, n (%)	2 (5.9)	1 (3.2)
Coronary artery disease, n (%)	10 (29.4)	10 (32.3)
Diabetes mellitus, n (%)	10 (29.4)	7 (22.6)
Peripheral vascular disease, n (%)	8 (23.5)	6 (19.4)
Medication		
ACEi/ARB, n (%)	18 (52.9)	15 (48.4)
Beta blocker, n (%)	21 (61.8)	19 (61.3)

*NYHA* new york heart association, *BMI* body mass index, *COPD* Chronic obstructive pulmonary disease, *NT-proBNP* N-terminal pro B-type natriuretic peptide, *LVEF* left ventricular ejection fraction, *ACEi* angiotensin-converting enzyme inhibitor, *ARB* angiotensin receptor blocker

Data are presented as mean ± SD or median (interquartile range)

**Fig. 2:**
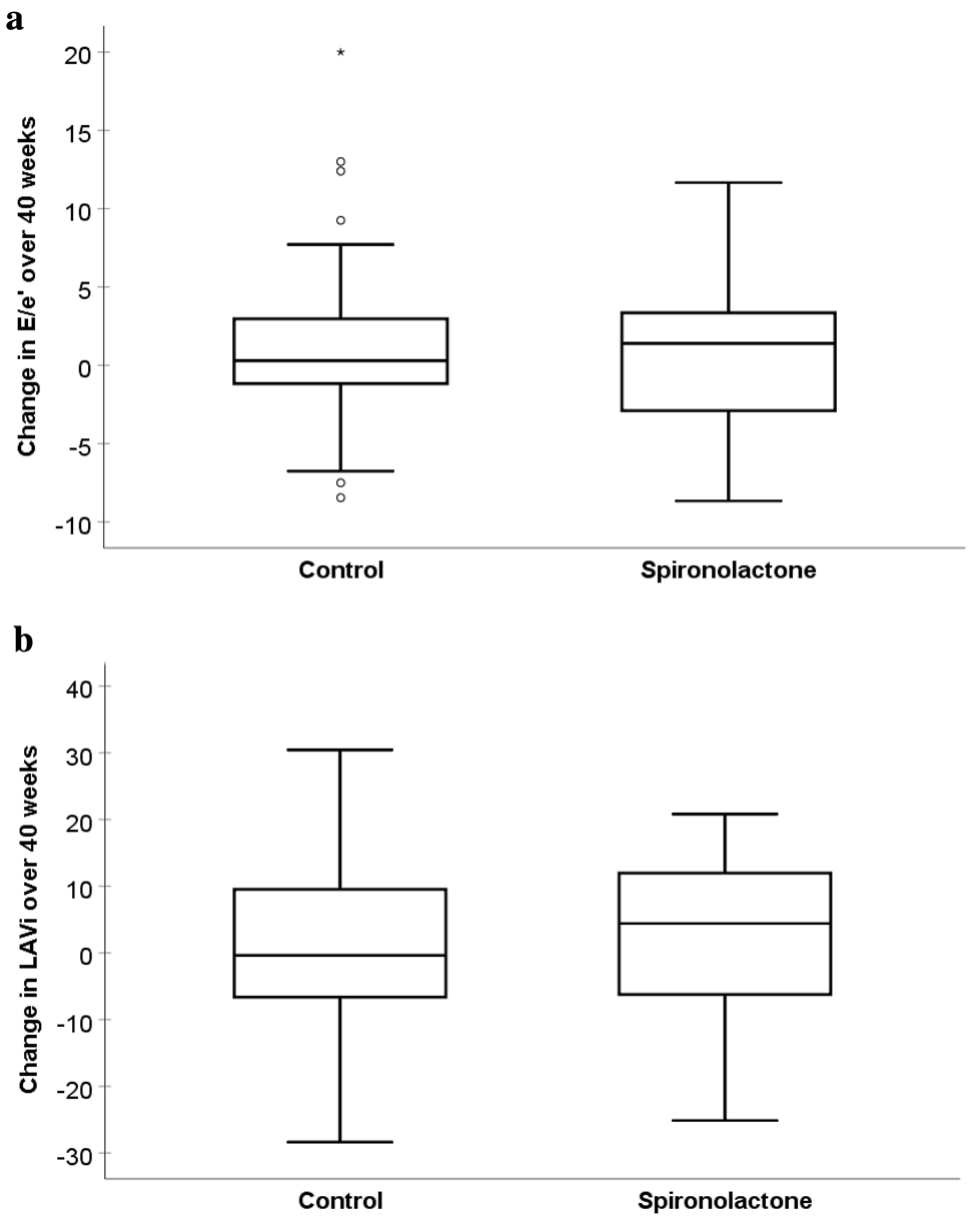
**a** Change in E/e’ after 40 weeks of treatment with spironolactone or placebo (p = 0.68) **b** Change in left atrial volume index (LAVi) after 40 weeks of treatment with spironolactone or placebo (p = 0.89) Whiskers represent 95% CI

**Fig. 3:**
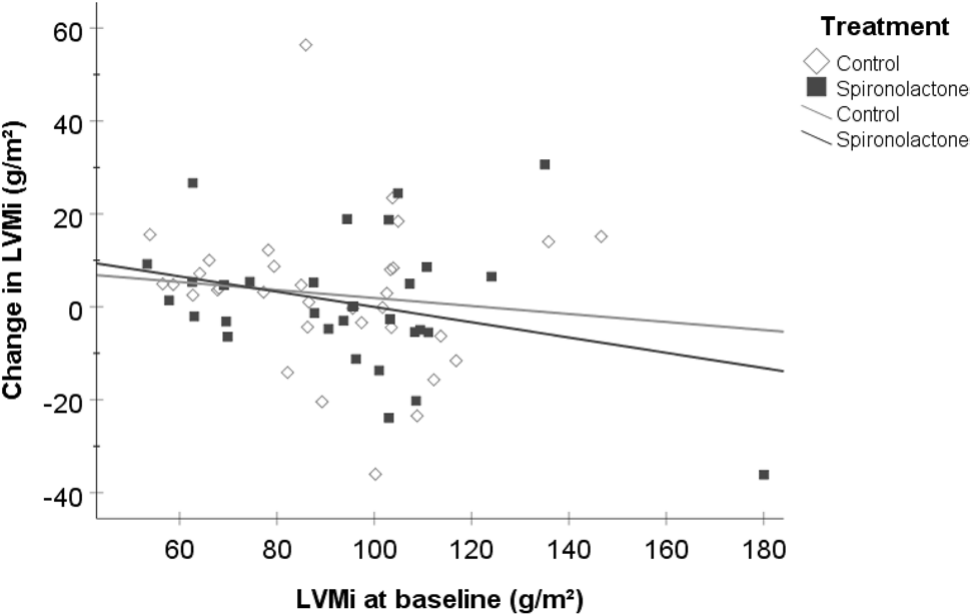
Mean change in LVMi by LVMi at baseline after 40 weeks of treatment with spironolactone compared to placebo (p = 0.72)

**Fig. 4:**
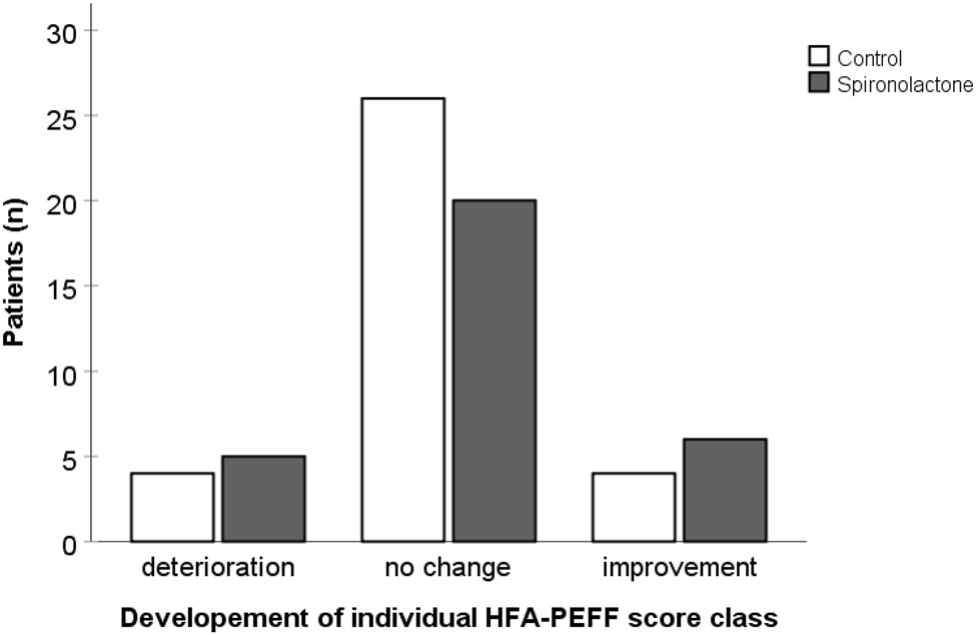
Changes in the HFA-PEFF score class after 40 weeks of treatment with spironolactone or placebo (p = 0.63)

There were no significant differences regarding the change in echocardiographic parameters between the two study groups (Table 2). After 40 weeks of treatment, there was no significant difference in mean change in E/e` (spironolactone vs. placebo: + 0.93 ± 5.39 vs. + 1.52 ± 5.94, p = 0.68, Fig. 2a) or mean change in LAVi (1.9 ± 12.3 vs. 1.7 ± 14.1 g/m^2^, p = 0.89, Fig. 2b) between the two treatment groups. Moreover, there was no significant difference in mean change in LVMi (+ 0.8 ± 14.2 g/m^2^ vs. + 2.7 ± 15.9 g/m^2^; p = 0.72, Fig. 3) or mean change in systolic function parameters (LVEF: + 1.1 ± 6.5% vs -0.8 ± 6.6%; p = 0.44; GLS: −0.71 ± 2.84% vs. + 0.55 ± 2.11%; p = 0.76). There also was no difference between the spironolactone and the placebo group regarding mean change of NT-proBNP (median (IQR): 278 (−1270–6400) vs. + 591 (−894–2040) pg/ml, p = 0.96). After 40 weeks of treatment, the number of patients with HFpEF according to the HFA-PEFF score remained unchanged in both groups (spironolactone group: n = 18, placebo group: n = 19, Table 3) and there was no significant change in patients allocated to different HFA-PEFF score classifications (p = 0.63, Fig. 4).

**Table 2:** Nine-months change in parameters of cardiac geometry and function

	Placebo (n = 34)		Spironolactone (n = 31)		Mean difference in change (95% CI)	p value*
	Baseline	Follow-up	Baseline	Follow-up		
LVM, g	177 ± 46	184 ± 50	192 ± 55	191 ± 51	4.4 (−9.8–18.6)	0.54
LVMi, g/m^2^	91 ± 22	94 ± 26	95 ± 26	95 ± 25	1.4 (−6.1–8.8)	0.72
LAVi, ml/m^2^	41.9 ± 14.6	43.5 ± 19.7	44.7 ± 21.1	44.8 ± 18.4	−0.5 (−7.1 – 6.2)	0.89
LVEF, %	66.3 ± 6.4	65.5 ± 6.3	64.7 ± 6.5	65.8 ± 7.4	−1.1 (−4.1–1.8)	0.44
LVEDD, mm	45.2 ± 5.7	46.1 ± 5.7	47.3 ± 5.5	47.0 ± 4.9	0.6 (−1.2–2.3)	0.52
GLS, %	17.9 ± 2.4	17.6 ± 2.3	16.2 ± 1.9	16.7 ± 3.0	−0.3 (−2.4–1.7)	0.76
A-Wave, cm/s	91.2 ± 26.5	94.9 ± 19.6	87.7 ± 25.5	88.1 ± 25.0	5.2 (−3.8–14.2)	0.25
E-Wave, cm/s	85.0 ± 26.5	94.1 ± 38.0	85.8 ± 44.1	91.2 ± 44.3	3.6 (−7.3–14.5)	0.51
e’, cm/s [septal]	6.3 ± 2.0	6.6 ± 2.3	5.9 ± 1.8	6.0 ± 1.6	0.3 (−0.5–1.1)	0.42
e’, cm/s [lateral]	8.8 ± 2.9	9.2 ± 2.4	9.1 ± 2.9	9.0 ± 2.4	0.2 (−0.9–1.3)	0.73
E/A	0.9 ± 0.3	1.0 ± 0.3	0.9 ± 0.4	1.0 ± 0.3	−0.1 (−0.2–0.1)	0.87
E/e’ [septal]	14.6 ± 6.2	16.1 ± 9.4	15.8 ± 9.3	16.7 ± 10.1	0.6 (−2.3–3.5)	0.68
RWT	0.47 ± 0.07	0.46 ± 0.07	0.45 ± 0.10	0.45 ± 0.09	−0.01 (−0.03–0.01)	0.37
DT, ms	248 ± 64	238 ± 44	269 ± 91	258 ± 75	−12 (−39–14)	0.36
IVRT, ms	107 ± 18	102 ± 18	109 ± 23	107 ± 22	−4 (−12–5)	0.41
TR velocity, m/s	2.5 ± 0.4	2.7 ± 0.5	2.5 ± 0.5	2.6 ± 0.6	0.1 (−0.1–0.3)	0.43

*DT* deceleration time, *GLS* global longitudinal strain, *IVRT* Isovolumetric relaxation time, *LAVi* Left ATRIAL volume index, *LVEDD* left ventricular end-diastolic diameter, *LVEF* left ventricular ejection fraction, *LVM* left ventricular mass, *LVMi* LVM index, *RWT* relative wall thickness, *TR* tricuspid valve regurgitation

Missing data: TR velocity (placebo group): n = 12, GLS (spironolactone group): n = 13; GLS (placebo group): n = 21, e’ [lateral] (placebo group): n = 13. All data except LAVi were collected using echocardiography, LAVi measurements are based on CMR imaging

^*^One-way ANCOVA analyses with adjustment for the respective baseline values

Data are presented as mean ± SD

**Table 3:** Distribution of HFA-PEFF score classification at baseline and follow-up

	Placebo		Spironolactone	
	Baseline	Follow-up	Baseline	Follow-up
“no HFpEF” (0–1 points)	0 (0.0%)	0 (0.0%)	0 (0.0%)	1 (3.2%)
“further testing required” (2–4 points)	15 (44.1%)	15 (44.1%)	13 (41.9%)	12 (38.7%)
“HFpEF” (5–6 points)	19 (55.9%)	19 (55.9%)	18 (58.1%)	18 (58.1%)

*HFpEF* Heart failure with preserved ejection fraction

Data are presented as n (%)

## Discussion

The main finding of this study is that treatment with 50 mg of spironolactone for 40 weeks had no significant effect on cardiac function, particularly diastolic function parameters in HD patients. In addition, the frequency of HFpEF according to the HFA-PEFF score was unchanged.

Several randomized-controlled studies reported conflicting results on the efficacy of spironolactone to achieve improvements regarding HFpEF or diastolic dysfunction (DD) in patients with no or minor kidney impairment. Our study provides additional insights on the usefulness of MRA in this matter in HD patients. The Aldo-DHF trial was first to show beneficial effects of spironolactone intake compared to placebo in patients suffering from DD with regard to E/e’, NT-proBNP and left ventricular mass after 12 months in patients with unimpaired kidney function (mean GFR: 79 ml/min/1.73m^2^) [10]. Further evidence of a favourable effect of spironolactone in HFpEF patients has been published by Kosmala et al*.* who observed that treatment with spironolactone improved exercise capacity in HFpEF patients and was associated with a reduction in exercise-induced increase in E/e’ when compared to placebo [23]. Focusing on female individuals suffering from HFpEF, Kurrelmeyer et al*.* found that six months of treatment with 25 mg of spironolactone vs. placebo had a beneficial effect on diastolic function parameters (e’, E/e’) as well as on a composite score considering change in NYHA functional class, change in results of the Kansas City Cardiomyopathy Questionnaire score, hospitalization for worsened HF and death [24]. In contrast, findings of Upadhya et al*.* showed that 25 mg of spironolactone did not improve exercise capacity or reduce LVM [25]. The only outcome trial investigating spironolactone in HFpEF so far, TOPCAT, found no improvement in hospitalization or cardiovascular death after 3 years of spironolactone treatment compared to placebo in 3,445 HFpEF patients [11]. The validity of the trial results was subsequently questioned due to the suspected suboptimal trial conduct in some participating countries [26]. Data on the effect of spironolactone in HD patients is very limited. Recently, Charytan et al*.* published the results of the Spin-D trial which investigated the effect of different dosages of spironolactone (12.5 mg, 25 mg or 50 mg) compared to placebo over 36 weeks in 129 ESKD patients. Here, spironolactone failed to improve cardiac function parameters and yielded no effect on E/e’, LVMi or GLS [12].

Although dyspnoea on exertion and apparent signs of diastolic dysfunction were not a prerequisite for inclusion into the MiREnDa trial, the majority of our patients exhibited increased mean LAVi and/or mean E/e’ at baseline indicating elevated left ventricular stiffness and increased left ventricular filling pressure [14, 16]. This reflects findings by Antlanger et al*.* who showed that out of 105 HD patients, 96% presented with signs of DD on examination and 57% fulfilled the diagnostic criteria of HFpEF [2]. Consistent with the Spin-D trial, we did not observe an effect of spironolactone on E/e’ or other parameters of diastolic function in the MiREnDa trial, even though all of our patients received a rather high dose of spironolactone when compared to other trials that observed a positive treatment effect [10, 24]. We implemented a follow-up period of nine months that might have been too short to detect changes in diastolic function that require prolonged treatment. However, there appears to be no relation between positive treatment effects and trial duration. We rather find trials with comparably short periods of observation (6–12 months) finding positive treatment effects in some cases [10, 23, 24] and the absence of such in other [12, 25]. Furthermore, given that diastolic function was not the primary focus of our study, a lack in power to detect small changes in diastolic function might be an explanation for our neutral result. At the same time and in line with our previously published data, there were no major discrepancies regarding dropouts between the two study arms [27]. Finally, since all above-mentioned studies apart from the Spin-D trial excluded patients with impaired kidney function, their results might not be directly transferable to an ESKD population. Especially patients on maintenance HD without residual renal function regularly present with different grades of volume overload and even though we attempted to account for fluid variations by scheduling all assessments on dialysis-free days approximately 24 h subsequent to the last HD session, most echocardiographic measurements remain vulnerable to volume status variations that occur in intermittent HD [28].

Spironolactone treatment also had no effect on LVMi as assessed by echocardiography which is consistent with our recently published CMR imaging data [27]. Since the prevalence of LVMi is known to be associated with poor diastolic function [15, 29], a similar treatment effect of spironolactone on both LVMi and diastolic function parameters was expected. Its absence regarding LVMi further validates our echocardiographic findings concerning DD. Noteworthy, the relatively small sample size and short follow-up period might have hindered the observation of a treatment effect. As only patients with preserved LVEF were included in this echocardiographic substudy of the MiREnDa trial, the effect of spironolactone in patients with reduced ejection fraction was not part of the analysis. We did, however, observe an impaired GLS at baseline that could be interpreted as an early and sensitive surrogate of systolic dysfunction. In a substudy of the TOPCAT trial, an impaired GLS was a strong predictor of cardiovascular adverse events and could be improved by spironolactone treatment in patients with HFpEF [30]. We did not see a comparable effect of treatment in the MiREnDa population after spironolactone treatment but were limited by a smaller sample size and shorter follow-up period.

In addition to the effect of spironolactone on individual echocardiographic parameters, we investigated the treatment effect on the HFA-PEFF score assuming it to be an even more sensitive means to detect treatment effects as the score integrates functional and morphological parameters as well as natriuretic peptides levels. Although the score was developed to aid the diagnosis of HFpEF in patients with dyspnoea of unknown cause, the absence of treatment effect compared to placebo further underlines our findings. Notably, in an HD context, shortness of breath on exertion is only one of the symptoms of heart failure, rather unspecific and particularly unreliable [2, 13].

### Limitations and strengths

There are some limitations to our study. Lateral e’ measurements were not broadly available throughout our study group. Since we focused on the change of E/e’ in the course of placebo or spironolactone administration rather than on absolute values, we did not consider this a major factor influencing our findings. Moreover, septal e’ has been reported to be less affected by HD than lateral or averaged e’ [28]. We used two-dimensional echocardiography instead of three-dimensional echocardiography to quantify cardiac structural parameters. Although this is in accordance with current guidelines [14, 15], edge cases with severe cardiac deformities (e.g. caused by previous large myocardial infarction) might be a source of inaccuracy. In order to account for the difference between LAVi measurements in CMR and echocardiography, we used adjusted cut-off values. This might be a source of inaccuracy possibly decreasing the ascertained number of patients diagnosed with HFpEF according to the HFA-PEFF algorithm. Since HD patients have markedly higher serum levels of NT-proBNP [31] and biomarker levels of NT-proBNP are one of the three diagnostic pillars in the HFA-PEFF score, the use of predefined NT-proBNP cut-off values might be another source of inaccuracy regarding the observed frequency of HFpEF. It remains unclear whether the observed elevated biomarker levels are only a sign of overhydration instead of actual cardiac impairment [32]. Moreover, female and non-Caucasian HD patients were underrepresented, therefore our findings might not be generalizable.

Our study has several strengths including the randomized, controlled trial design, applying a relatively high dosage of spironolactone and analysing parameters of diastolic function that were predefined as secondary endpoints at the time of trial commencement. Additionally, to ensure image quality and reproducibility imaging data was evaluated in a core laboratory, readers were blinded to treatment allocation and in the event of predefined signs of poor image quality or challenging echocardiographic conditions multiple imaging runs per patients were performed.

## Conclusion

In summary, 40 weeks of spironolactone treatment did not change diastolic function parameters or impact HFA-PEFF score classifications in HD patients compared to placebo.

## Data Availability

The data generated during the MiREnDa trial are not publicly available due to data protection and privacy. The datasets can be made available on request.
